# Determinants of depression and anxiety among type 2 diabetes patients in governments’ hospitals at Harari regional state, Eastern Ethiopia: A multi-center cross-sectional study

**DOI:** 10.1186/s12888-022-04494-x

**Published:** 2023-01-05

**Authors:** Kabtamu Nigussie, Addisu Sertsu, Galana Mamo Ayana, Yadeta Dessie, Tilahun Bete, Lemesa Abdisa, Gebiso Roba Debele, Dawud Wadaje, Abraham Negash

**Affiliations:** 1 Department of Psychiatry, College of Health and Medical Sciences, Haramaya University, Harar, Ethiopia; 2 Department of Nursing, College of Health and Medical Science, Haramaya University, Harar, Ethiopia; 3 Department Epidemiology and Biostatistics, School of Public Health, College of Health and Medical Science, Haramaya University, Harar, Ethiopia; 4 Department of Public Health, College of Health and Medical Science, Haramaya University, Harar, Ethiopia; 5 Department of Public Health, College of Health Science, Mettu University, Mettu, Ethiopia; 6 Department of Midwifery, College of Health and Medical Science, Haramaya University, Harar, Ethiopia

**Keywords:** Type 2 diabetic mellitus, Determinants, Depression, Anxiety, Magnitude, Out-patient, Ethiopia

## Abstract

**Background:**

Type 2 diabetes mellitus is the most common health problem globally. Depression and anxiety can exacerbate disease complications, make patients suffer more, and increase healthcare costs. Even though, depression and anxiety are common among type 2 diabetes mellitus patients, there have been limited studies conducted about the determinants of depression and anxiety in Ethiopia. Therefore, the purpose of this study was to assess the magnitude and determinants of depression and anxiety symptoms among Type 2 diabetes mellitus patients, attending out-patient treatment at Harari regional state government hospitals, Eastern Ethiopia.

**Method:**

An institutional based cross-sectional study was conducted from March to April at Harari regional state government hospitals in eastern Ethiopia. A total of 421 participants were recruited using the systematic sampling technique. Data was collected by using Afan Oromo version of interviewer-administered structured and semi-structured questionnaires. Depression and Anxiety symptoms were assessed by the Hospital Anxiety and Depression Scale. Bivariate and multivariate logistic regression analysis was done to identify variables related to both depression and anxiety symptoms. The association was described using an adjusted odds ratio and a 95% confidence interval (CI), with P-values of 0.05 used as a cutoff for a significant association in the adjusted analysis.

**Result:**

Out of the 416 participants included in this study, 42.3%, 40.4% had depression and anxiety symptoms, respectively. Being female (Adjusted Odds Ratio = 1.85(1.09–3.15)), no formal education (Adjusted Odds Ratio = 2.65, (1.04–6.73)), age ≥ 70 (Adjusted Odds Ratio = 2.88 (1.28–6.48)), family history of mental illness (Adjusted Odds Ratio = 1.71 (1.35–3.82)) and poor social support (Adjusted Odds Ratio = 2.35(1.12–6.03)) were statistically associated with depression. While having a family history of mental illness (AOR 1.74(1.03–2.95)), being widowed (AOR = 3.45(1.49–8.01)), and having poor social support (AOR = 2.15(1.12, 4.89)) were statistically significant associated with anxiety at a *p*-value < 0.05.

**Conclusion:**

Current study results showed that the magnitude of depression and anxiety were relatively high among type 2 diabetes mellitus patients.Having a family history of mental illness and poor social support were statistically associated with both depression and anxiety symptoms. Screening, early detection, and appropriate treatment of depression and anxiety symptoms in type 2 diabetes mellitus patients should be prioritized by health care providers.

## Background

Diabetes mellitus is a chronic metabolic disease characterized by decreasing insulin secretion or due to reduction insulin sensitivity of the cell of the body cell [1]. The International Diabetes Federation (IDF) has released new figures showing that 537 million adults are now living with diabetes worldwide a rise of 16% (74 million) since the previous IDF estimates in 2019. International Diabetes Federation (IDF) projections show that by 2045, 783 million adults will be living with diabetes – or one in eight adults. This would be an increase of 46%, more than double the estimated population growth (20%) over the same period [2, 3]. Type 2 diabetes Mellitus (T2DM) is one of a common health problem with serious medical and economic consequences. It is the third leading cause of disease burden in the worldwide [4]. According to the International Diabetes Federation (IDF), the magnitudes of type 2 diabetes mellitus in Ethiopia is steadily increasing with 3.5% in 2011, 4.36% in 2013, and 7.4% in 2015 making one of the top four countries with the highest adult diabetic populations in sub-Saharan Africa [5, 6].

The co-occurrence, of biological, psychological and psychosocial issues can complicate the diagnosis and management of diabetes mellitus [7]. Depression is common co-morbid conditions that exacerbate the progression of diabetes-related complications and more doubles the risk of depression compared to the general population and It has been reported that depression is twice as common in individuals with type 2 diabetes [8–10]. More than fifty percent (52.5%) of patients who had DM for > 20 years were depressed [11, 12]. A growing evidence showed that depression among type 2 diabetes mellitus patients worsen adherence to dietary, medication, physical inactivity and metabolic control which intensify diabetic complications, disease severity and increases health-care expenditures, there by lowers patients’ quality of life [13].

Anxiety is subjective, a vague, non-specific feeling of uneasiness, apprehension, tension, excessive nervousness, and a sense of impending doom, irrational avoidance of objects or situation and anxiety attack [14]. The anxiety disorders make up one of the most common groups of psychiatric disorders and the national co-morbidity study reported that one of four persons met the diagnostic criteria for at least one anxiety disorder and presence of anxiety symptoms significantly impairs the health related quality of life among those with diabetes type 2 mellitus patients [15].

People with diabetes are twice as likely to be diagnosed with anxiety and depression than those without diabetes [11, 16]. Different studies revealed that gender, age, education, diabetes complications and poor glycemic control were related to anxiety and depression in people with T2DM [11, 16–18]. Comorbid depression and anxiety are associated with negative health outcomes, such as increased diabetes-related complications [17]**,** increased physical morbidity, higher blood glucose levels [11, 16], poor quality of life [19] and premature death, as compared with depression or anxiety alone [20]. Poor glycemic control and functional impairment due to increasing diabetes complications may cause or worsen depression and anxiety in patients [9, 21, 22].

Despite the fact that 80% of people with type 2 diabetes mellitus live in low and middle-income countries, many studies on depression and anxiety symptoms among type 2 diabetes mellitus patients were conducted in the developed world [22]. Moreover, only a few studies have been conducted in Africa, particularly in Ethiopia. There is a little data about determinants of depression and anxiety among type 2 diabetes mellitus patients which has become the main problem for intervention to reduce the impact of depression and anxiety among type 2 diabetes mellitus patients, and there has been limited study done on the prevalence of depression and anxiety among comorbid type 2 diabetes mellitus patients in Ethiopia, including our study area. Therefore, the aim of this study is to determine of the magnitudes and determinants depression and anxiety symptoms among type 2 diabetes mellitus patients attending out-patient treatment at government hospitals in Harari regional state, Eastern, Ethiopia.

## Methods and materials

### Study design, setting and period

An institutional-based cross-sectional study design was conducted in Harari regional state among three government hospitals, which are Hiwot Fana Specialized University Hospital, Jugal, and Harari Federal Police Hospital, from March to April 2022. According to a Harari Regional Health Office report, approximately 25,000 diabetes patients were recently receiving follow-up care. Every month, approximately 873 type 2 diabetes mellitus patients visit Hiwot Fana Specialized University Hospital, as well as Jugal and Harari Federal Police Hospitals, for follow-up.

### Eligible criteria

All adults (age ≥ 18 years) with type 2 diabetes mellitus patients, who were in an out-patient treatment care of the hospitals during the study period, were included in the study. Patients who had critical health conditions (uncertain prognosis, vital signs are unstable, there are major complications) and those patients unable to communicate properly were excluded.

### Sample size determination and sampling procedure

The adequate sample size was calculated by using a single population proportion formula with the following statistical assumptions: *n *= the minimum sample size required, *p* = the estimated proportion of depression, z = the standard value of a confidence level of alpha = 95%, d = the margin of error between the sample and the population (0.05). For this study *p* = 47%, which is the magnitude of depression among type two diabetes mellitus patients in southwest Ethiopia [15].

Accordingly, adding 10% for the non-response rate gives the total calculated sample size of 421. Then, the number of patients was proportionally allocated among the government hospitals. Finally, eligible patients were selected using a systematic random sampling technique with K-value of 2 after proportional allocation was completed for each hospital, K = N/n, 873/421≈2 with a k = 2. The first study participant was selected by a lottery method (manual lottery) from each hospital independently, and then the next study participants were selected at a regular interval (every two) individual.

### Variables of study and measurements

#### Dependent variable

Status of depression symptoms (yes/no) and anxiety symptoms (yes/no).

#### Independent variables

Socio-demographic variables (sex, age in years, marital status, religion, occupational status, educational status, residence, and average monthly income in Ethiopian birr); clinical factors (family history of mental illness, family history of diabetes mellitus, diabetic complications, Body mass index, Age onset of illness, glycemic control, physical activity, and comorbid medical illness), substance-related factors (current and lifetime substance use of alcohol, tobacco, Khat, and cannabis/ hashish); and psychosocial factors (social support).

#### Data collection instruments and procedure

Face-to-face interviews were used to collect data using structured and semi-structured questionnaire and a review of patients’ charts. Sociodemographic characteristics’ of participants were collected by structured questionnaire that adapted from different literature. All variables were categorical except age which was collected by close ended questionnaire. Depression and Anxiety was assessed by Hospital anxiety and depression scale. Hospital anxiety and depression scale was used and validated in Ethiopia with internal consistence of 0.78, for anxiety sub-scale 0.76, for depression sub-scale and 0.87 for the full version hospital anxiety and depression scale. It is commonly used to screen for anxiety and depression symptoms, and it has 14 item questions which are divided into two parts, which is a 7- item sub-scale for each depression and anxiety symptoms. The items are rated on a four-point Likert scale which ranges from 0 to 3 with maximum and minimum score of 0 and 21 receptively. If the participant scores ≥ 8 for each of the depression and anxiety sub-scale questions, were considered as the participant has anxiety and depression, respectively [23].

Social support was assessed by Oslo social support scale (Oslo-3) containing three items. Social support was collected by the Oslo-3 item of the social support scale. It is a 3 item questionnaires, commonly used to assess social support and it has been used in several studies. The sum score scale ranged from 3–14, which had 3 categories: poor support 3–8, moderate support 9–11, and strong support 12–14 [24]. It was used in different study and validated in Ethiopia [25].

Substance-related factors were assessed by the Alcohol, Smoking, and Substance Involvement Screening Test(ASSIST), which is a brief screening questionnaire developed and validated by the world health organization (WHO) to find out about people’s use of psychoactive substances was used to assess current and ever substance use history of the subject [26].

Glycemic control was defined according to American Diabetes Recommendation Venus Blood draw for measurement of Glycated hemoglobin (HbAlc). Poor glycemic control was evaluated (HbAlc: < 7% versus ≥ 7% whereas good glycemic control, if patients score 70–130 mg/dl of fasting blood glucose upon measurement of three consecutive visits [27–29].

Physical activity**:** Physical activity was assessed by 2 items of days in the last seven day in week. Then, the responses were added up (range, 0–14). Participants who scored ≥ 8 were coded as adhering to the physical activity recommendations [30].

#### Data quality control

Data collectors and supervisors were trained for four days on the data collection approach of the study. The questionnaire was translated into local language, Afan Oromo, by language experts and back-translated into English by another person to check for consistency. A pretest was conducted on 21(5%) of the sample size at Haramaya General Hospital to see the applicability of the instruments, and feedback was incorporated into the final tool to improve the quality. The result was not included in the results of this study. Collected data was checked daily for completeness and consistency of filled questionnaires. All data collectors and supervisors were adherent to COVID-19 prevention protocol.

#### Data processing and analysis

The data were coded, cleaned, and entered into Epi Data version 4.6.0.2 which was then exported to SPSS (Statistical Package for Social Science) version 20 for analysis. Bivariable and multivariable logistic regression analysis was performed to identify factors associated with an outcome variable. All variables with a p-value less than 0.05 in bivariable analysis were entered into the multivariable logistic regression analysis. A p-value of < 0.05 was considered statistically significant, and the adjusted odds ratio (AOR) with a 95% confidence interval (CI) was calculated. The goodness of fitness was checked by the Hosmer-Lemshow test.

#### Ethical consideration

The study was carried out under consideration of the Helsinki Declaration of medical research ethics [31] .Ethical clearance was obtained from the Institutional Health Research Ethics Review Committee (IHRERC) of Haramaya University College of Health and Medical Sciences with reference number IHREREC/060/2022. A formal letter of permission and support was provided to all three government hospitals in which the study was conducted. Informed, voluntary, written, and signed consent was obtained from the heads of the respective hospitals. Participants were informed about the aim of the study and the advantage of the study; confidentiality, there was no risk of being participants, and they had full right to stop in the middle of the interview. Oral and written informed consent was taken from each participant before data collection began. Confidentiality was maintained at all levels of the study through anonymous data collection. During data collection, the COVID-19 prevention protocol was strictly followed.

## Results

### Socio-economic characteristics of participants

Out of the 421 study participants, 416 patients were recruited in the study with response rate (98.8%). The median age of the respondents was 53.5 with inter quartile range (IQR, 45–62) years. Among respondents, around half218 (52.4%) were male, and three-fourth, 314 (75.5%) were married. Nearly one in three, 133(32%) of study participants were self-employed and attended secondary school [9–12] education while most of them 346 (83.2) and 394 (94.7%) were live in urban and with their families respectively, as shown below in (Table 1).

**Table 1:** Socio-demographic characteristics of patients with type 2 diabetes mellitus attending Government hospitals in Harari regional state, East Ethiopia (*n* = 416)

Variable	Categories	Frequency	Percent (%)
Age	18–39	63	15.1
	40–49	96	23.1
	50–59	119	28.6
	60–69	93	22.4
	≥ 70	45	10.8
Sex	Male	218	52.4
	Female	198	47.6
Religious	Muslim	181	43.5
	Orthodox	175	42.1
	Protestant	48	11.5
	Others*	12	2.9
Marital status	Married	314	75.5
	Single	24	5.8
	Divorce	34	8.2
	Widowed	44	10.6
Educational level	No formal education	65	15.6
	Primary (1–8)	103	24.8
	Secondary (9–12)	133	32
	Diploma	43	10.3
	Degree and above	72	17.3
Jobs	Government worker	118	28.4
	Self employed	133	32
	Unemployed	31	7.5
	Student	9	2.2
	Farmer	59	14.2
	Housewife	46	11.1
	Retirement	20	4.8
Residence	Urban	346	83.2
	Rural	70	16.8
Living arrangement	With family	394	94.7
	Alone	22	5.3
Income monthly in Ethiopian birr (ETB)	< 1000	41	9.9
	1000–5000	153	36.8
	5001–10,000	134	32.2
	> 10,000	88	21.2

^*^Others: Catholic, Wakefata and Adventist and 1EBT = 0.019 United State Dollar

### Clinical, substance use and psychosocial characteristics of the respondents

Out of 416 participant, 134(32.2%) of the respondents had up to one year duration of illness whereas 55 (13.2%) of the study participants had complication of diabetes mellitus. From all study participants 130(31.2%) had good glycemic control, 154 (37.0%) had normal body mass index whereas 336 (80.8%) of participants had regular physical activity. Around one in three study participants 133(32.0%) and 134(32.2%) were use only insulin for diabetic treatment and 50–59 age range while disease onset. Nearly half of study participants, 215 (51.7%) have strong social support and 234(56.3%) were ever Khat user as shown below in (Table 2).

**Table 2:** Clinical, substance use and psychosocial characteristics of patients with type 2 diabetes mellitus attending Government hospitals in Harari region state, East Ethiopia (*n* = 416)

Variable	Categories	Frequency	Percentage
Duration of illness in year	Up to 1 year 1–5 6–10 ≥ 10	134 130 118 34	32.2 31.3 28.4 8.2
Family history of T2DM	Yes No	172 244	41.3 58.7
Family history mental illness	Yes No	116 300	27.9 72.1
Diabetic complications	Yes No	55 361	13.2 86.8
Body mass index in kg	Normal Underweight Overweight Obesity	154 61 114 87	37.0 14.7 27.4 20.9
A drug used for diabetes	Insulin only Single OHA* Combination of OHA* Insulin with OHA*	133 35 59 189	32.0 8.4 14.2 45.4
Age starts of disease in year	< 40 40–49 50–59 ≥ 60	102 112 134 68	24.5 26.9 32.2 16.3
Glycemic control	Good Poor	130 286	31.2 68.8
Physical activity	Yes No	336 80	80.8 19.2
Ever alcohol use	Yes No	97 319	23.3 76.7
Ever cigarette smoking	Yes No	63 353	15.1 84.9
Ever Khat use	Yes No	234 182	56.3 43.8
Current Alcohol use	Yes No	55 361	13.2 86.8
Current cigarette use	Yes No	39 377	9.4 90.6
Current Khat use	Yes No	179 237	43 57
Social support	Poor	33	7.9
	Moderate	168	40.4
	Strong	215	51.7

^*^OHA =Oral hypoglycemic agent

### Factors associated with depression among patients with type 2 diabetes mellitus

In Bivariate logistic regression analysis, age ≥ 70, being female, divorced, widowed, no attend formal education, monthly income less than 1000, having family history of mental illness, having family history of DM, age of start disease less than 50 years,, ever substance use, ever Khat use, poor and moderate were factors significantly associated with depression. However, in the multivariate logistic regression analysis, variables like age ≥ 70, being female, having no formal education, having monthly income less than 1000 EBT, having family history of mental illness, and having poor social support were significantly associated with depression at a p value of 0.05.

In this study, the odds of having depression among respondents with age ≥ 70 were about 2.88 times higher as compared to participants those age range 18–39 (AOR = 2.88 (1.28–6.48)), and the odds of having depression among female participants was 1.85 times higher as compared to male respondents (AOR = 1.85(1.09–3.15)).

Participants who did not attend formal education had 2.65 times the odds of having depression as those who had a degree or higher educational status (AOR = 2.65, (1.04–6.73)). The findings of this study indicated that the odds of having depression among participants who had family history of mental illness and poor social support were about 1.71 and 2.35 times higher as compared to participants who had no family history of mental illness and had strong social support (AOR = 1.71 (1.35–3.82)), (AOR = 2.35 (1.12–6.03)) respectively as shown in the table below (Table 3).

**Table 3:** Factors associated with depression among patients with type 2 diabetes mellitus attending government hospitals in Harari regional state East Ethiopia (*n* = 416)

Variable	Categories	Depression(*n* = 416)		COR (95% CI)	AOR(95% CI)
		No	Yes		
Age in year	18–39	50	13	1	1
	40–49	58	38	2.40(1.17–4.92)	1.98(0.90–4.35)
	50–59	67	52	2.63(1.31–5.27)	2.22(0.90–6.62)
	60–69	46	47	2.76(1.34–5.68)	2.26(0.93–5.36)
	≥ 70	19	45	4.79(2.07–11.08)	2.88(1.28–6.48)**
Sex	Male	139	79	1	1
	Female	101	97	3.69(1.14–2.50)	1.85(1.09–3.15)*
Marital status	Married	201	113	1	1
	Single	14	10	1.27(0.52–2.95)	1.17(0.44–3.07)
	Divorce	13	21	2.83(1.39–5.96)	1.60(0.64–3.97)
	Widowed	17	27	2.85(1.48–5.41)	1.22(0.54–2.80)
Educational level	No formal education	33	32	2.87(1.45–5.66)	2.65(1.04–6.73)*
	Primary (1–8)	52	51	0.80(0.44–1.47)	1.01(0.46–2.21)
	Secondary (9–12)	90	43	0.90(0.51–1.59)	1.85(0.85–4.00)
	Diploma	28	15	0.60(0.31–1.17)	0.84(0.38–1.86)
	Degree and above	37	35	1	1
Income monthly	< 1000	9	32	5.14(2.19–12.05)	4.04(1.03–13.27)*
	1000–5000	93	60	0.93(0.95–1.59)	1.25(0.59–2.62)
	5001–1000	86	48	0.81(0.46–1.40)	0.86(0.44–1.83)
	> 10,000	52	36	1	1
Family history of DM	Yes	88	84	1.58(1.06–2.34)	1.08(0.64–1.83)
	No	152	92	1	1
Family history mental illness	Yes	53	63	2.15(1.42–3.27)	1.71(1.35–3.82)*
	No	187	113	1	1
Age starts of disease	< 40	70	32	0.32(0.17–0.61)	0.73(0.18–2.87)
	40–49	73	39	0.34(0.20–0.70)	0.52(0.16–1.67)
	50–59	69	65	0.66(0.37–1.20)	0.67(0.26–1.76)
	≥ 60	28	40	1	1
Ever Khat use	Yes	123	111	1.62(1.09–2.42)	1.00(0.46–2.13
	No	117	176	1	1
Social support	Poor	12	21	3.40(1.59–7.30)	2.35(1.12–6.03) **
	Moderate	86	82	1.86(1.23–2.81)	1.85(0.69–3.05)
	Strong	142	73	1	1

*= *p* < 0.05, and ** = *p* < *0.001, Chi square* = *9.70; DF* = *8 Hosmer-Lemshow test* = *0.43 COR*  *Crude odds ratio, AOR *Adjusted Odds Ratio

### Factors associated with anxiety among patients with type 2 diabetes mellitus

In bivariate logistic analysis, age greater than 40, divorced,widowed, attending secondary school (grade 9–12), monthly income less than 1000, greater than one year’s duration of illness, having family a history of mental illness, ever using Khat, and having moderate and poor social support were factors significantly associated with anxiety. However, multivariate analysis indicated, being widowed, family history of mental illness and poor social support were significantly associated with anxiety at a *p* value of 0.05.

In this study, participants with a family history of mental illness had 1.74 times the odds of having anxiety as those who did not have a family history of mental illness (AOR: 1.74 (1.03–2.95)). Odds of having anxiety among participants with being widowed were 3.45 times higher as compared to those being married participants (AOR = 3.45(1,49–8.01)) and odds of having anxiety among participants who had poor social support were 2.15 times higher as compared to those who had strong social support (AOR = 2.15, 95% CI: 1.12, 4.89) as shown below in (Table 4).

**Table 4:** Factors associated with anxiety among patients with type 2 diabetes mellitus attending government hospitals in Harari regional state East Ethiopia (*n* = 416)

**Variables**	**Categories**	**Anxiety** (*n* **= 416)**		**COR(95% CI)**	**AOR(95% CI)**
		**No**	**yes**		
Age in year	18–39	51	12	1	1
	40–49	62	34	2.33(1.10–5.00)	1.86(0.02–7.04)
	50–59	70	49	2.98(1.44–6.16	2.83(0.82–9.77)
	60–69	47	46	4.16(1.97–8.80)	3.44(0.81–14.63)
	≥ 70	18	27	6.38(2.68–15.17)	2.32(0.35–15.17)
Marital status	Married	205	109	1	1
	Single	14	10	1.34(0.58–3.13)	1.44(0.56–3.70)
	Divorced	16	18	2.12(1.04–4.31)	1.30(0.57–2.30)
	Widowed	11	33	5.64(2.74–11.00)	3.45(1.49–8.01)*
Educational status z	No formal education	309	35	1.18(0.60–2.28)	0.76(0.21–2.71)
	Primary (1–8)	63	40	0.46(0.35–1.68)	0.38(0.13–1.13)
	Secondary (9–12)	96	37	0.39(0.21–0.70)	0.41(0.15–1.17)
	Diploma	23	20	0.87(0.41–1.85)	1.20(0.50–2.88)
	Degree and above	36	36	1	1
Monthly income in Ethiopian Birr (ETB)	< 1000	18	28	3.42(1.56–7.50)	2.03(0.60–6.79)
	1000–5000	98	55	0.89(0.52–1.53)	1.22(0.66–2.48)
	5001–1000	83	51	0.98(0.56–1.70)	1.20(0.61–2.33)
	> 10,000	54	34	1	1
Duration of illness in years	Up to 1 year	94	40	1	1
	1–5	75	55	1.72(1.04–2.86)	1.28(0.71–2.32)
	6–9	60	58	2.27(1.36–3.81)	1.52(0.80–2.90)
	≥ 10	19	15	1.86(0.86–4.01)	2.12(0.69–6.51)
Family history mental illness	Yes	55	61	2.10(1.38–3.17)	1.74(1.03–2.95)*
	No	193	107	1	1
Ever Khat use	Yes	127	107	1.67(1.19–2.50)	1.26(0.77–2.07)
	No	121	61	1	1
Social support	Poor	13	20	3.00(1.41–6.36)	4.01(1.84–8.74)*
	Moderate	93	75	1.57(1.04–2.38)	1.21(0.74–1.96)
	Strong	142	73	1	1

^*^ = *p* < 0.05, and ** = *p* < *0.001, Chi square* = *7.80; DF* = *8 and Hosmer-Lemshow test* = *0.75*

## Discussion

This study finding show that significant number, 42.3% and 40.4% of type 2 diabetes mellitus patients had depression and anxiety symptoms respectively. This prevalence might be due impact of physical illness, comorbid innless and COVID-19. Having family history of mental illness and poor social support were statistically associated with both depression and anxiety.

The magnitude of depression symptoms among type 2 diabetic mellitus patients was 42.3% (95% CI, 37.7–47.1). This finding was in line with the study conducted in Mexican city 39% [32], South African 46% [33], Sudan 44% [34] and 47% at Ambo general hospital south west Ethiopia [15].

However, the result of this study,42.3% of depressive symptoms was lower than the study conducted in Nepal 57.8% [35], southern Iran 59% [36], Xuzhou, China 56.1% [37] and 47.9% Faisalabad [38]. The possible reason for the discrepancy might be due to the assessment tools for depression, Hospital Anxiety and Depression Scale (HADS) was used in this study but the study done in Nepal was used the Patient Health Questionnaire-9 (PHQ- 9) [35], Beck Depression Inventory in southern Iran [36], Depression Anxiety and Stress scale [38] and the Zung Self-Rating Depression Scales was used in China [37]. Other possible reason might be study design, an instructional based cross-sectional study was used in this study but study done in China and southern Iran were used community based cross-sectional and case–control respectively and also the study participants, environmental factors, sociocultural difference and age of participant.

On the other hand, the finding of current study was higher than the study done in Lithuania 22.4% [18], Kenya 32.3% [39] Sudan 35.6% [40] and south west Ethiopia 34.9% [41]. The possible reason for the discrepancy might be instrument used, which was in this study used Hospital Anxiety and Depression Scale (HADS), but the study done in Kenya [39], was used the Patient Health Questionnaire-9 (PHQ- 9), Beck Depression Inventory (BDI) scale questionnaire was used in southwest Ethiopia [41] and Patient Health Questionnaire-9 (PHQ- 9) was used in Eastern Sudan [40]. Study participants, setting and sociocultural difference might be also another possible reason for the discrepancy.

The prevalence of the anxiety symptoms among type 2 diabetic patient in this study was found to be 40.4%( 95% CI 35.6–45.4). This study result was in line with the study conducted in Saudi Arabia 45.45% [42], China 43.5% [37] and at Ambo General Hospital, southwest Ethiopia 44.2% [15].

However, the result of this study,40.4% Anxiety symptoms was lower than the studies conducted in Nepal 49.7% [35], southern Iran 62% [36], Faisalabad 69.6% [43] and Saudi Arabia 51.3% [44]. The possible reason for the discrepancy might be due to data collection tools for anxiety, which was this study used Hospital Anxiety and Depression Scale (HADS) but the study done in Nepal was used the Generalized Anxiety Disorders (GAD-7), Hamilton Anxiety questionnaires in southern Iran [36], Depression Anxiety and Stress scale (DASS) in Faisalabad [43]. The other possible reason for discrepancy might be the study participants, sample size and sociocultural difference of participant.

However, the finding of this study was higher than the studies conducted in Lithuania [18], North India 27.6% [45] and south Africa 32% [33]. The possible reason for variation might be due study design which was this study used cross-sectional study but cohort study was used south Africa [33] and instrument used which was this study used Hospital Anxiety and Depression Scale (HADS) but northern India [45] used Hamilton Anxiety Rating Scale.

Regarding factors associated with depression among type 2 diabetic patients, age ≥ 70 (older) of participants was significantly associated with depression. This finding was supported by studies conducted in Nepal [35], Xuzhou, China [37], South African [33], Kenya [39], and at Ambo general hospital [15]. This could be due to physical inactivates among elder patients. It is the fact that physical activity is a protective barrier against depression and the development of other psychological illnesses due to an decrease release of endorphins and brain neurotransmitters during exercise [46]. The other possible explanation is older patients faces many challenges including family or social isolation, different co-morbid diseases and disabilities.

The odds of having depression symptoms among respondents with female participants were 1.85 times higher when compared to participants with male gender. This result was supported by a studies conducted in Mexican [32], Nepal [35], China [37], South Africa [33],Sudan [40]and southwest Ethiopia [41]. The possible justification female were grater vulnerable to other psychosocial in additional to hormonal effect [8, 46]. Other possible reason might be related to depression are more common or two times as compared to male [46].

Regarding to family history of mental illness, participants who had family history of mental illness was 1.71and 1.74 times to had depression and anxiety respectively as compared to those who had no family history of mental illness. This was congruent with study carried out in Nepal [35] the possible reason might be family history of mental disorders are one the risk factor for mental disorder (the genetic effect) especially mood disorders which is if one parent has a mood disorder, a child will have a risk of between 10 and 25 percent for mood disorder [46].

The odds of having depression and anxiety symptoms among respondents with poor social support were 2.35 and 4.01 times higher as compared to those who had strong social support respectively. This finding was similar with prior studies conducted in Nepal [35], southwest Ethiopia [41] and at Ambo general hospital [15]. This might be a feeling of unsupported (isolated) and somatic illness (like diabetes mellitus) leads to increase psychosocial stress, in contrary good social support is critical for those in good health in reducing the risk of depression [47].

The odd of having anxiety symptoms among participants with being widowed was 3.45 times higher as compared to participants those being married. This finding was line with the studies conducted in Saudi Arabia [44] and South African [33]. The possible justification could be, widowed people might be experience anxiety symptoms like feelings of hopelessness, and worthlessness due to a lack of social support from their wife or husband.

### Limitations of the study

The retrospective items used in the questionnaire may have incurred recall bias, like duration of illness, duration of treatment, and types of medication. The cause-effect relationship between outcome variables and predictive variables was difficult to clarify because of the cross-sectional nature of the study. The COVID-19 pandemic might also affect the results of this study.

### Future directions

Based on the limitations of this study, we recommend that interested researchers in the fields of mental health and public health conduct longitudinal studies to investigate the magnitude of depression and anxiety symptoms. Furthermore, longitudinal studies are recommended to explore the cause-effect relationship between outcome variables and predictive variables.

## Conclusions

The findings of this study showed that the magnitude of depression and anxiety symptoms was relatively high, according to global estimation, among type 2 diabetes mellitus patients. Being female, age ≥ 70, not attending formal education, having average monthly income < 1000 EBT, having a family history of mental illness, and having poor social support were statistically associated with depressive symptoms whereas being widowed, having family history of mental illness and poor social support were statistically associated with anxiety symptoms. An effort should be taken by health care workers to strengthen and disseminate health education programs for diabetic patients and screening, early identification, and providing appropriate intervention for depression and anxiety among type 2 diabetes mellitus patients should be a great concern for the health care providers. It is better to establish health care programs, assessments and monitoring for people with type 2 diabetes mellitus who have a family history of mental illness, poor social support and patients will be treated early as soon as early identified. Intervene for this identified determinant was important for halt the distribution of depression and anxiety and reduce the complication of type 2 diabetes mellitus.

## Data Availability

The original contributions presented in the study are included in the article.

## Abbreviations

T2DM: Type 2 diabetes mellitus
HADS: Hospital Anxiety and Depression Scale
ASSIST: Alcohol, Smoking and Substance Involvement Screening Test
WHO: World health organization
